# Heterogeneity in general practitioners’ preferences for quality improvement programs: a choice experiment and policy simulation in France

**DOI:** 10.1186/s13561-016-0121-7

**Published:** 2016-09-15

**Authors:** Mehdi Ammi, Christine Peyron

**Affiliations:** 1 School of Public Policy and Administration, Carleton University, River Building, 1125 Colonel By Drive, Ottawa, ON K1S 5B6 Canada; 2 Laboratoire d’Économie de Dijon, Université de Bourgogne, CNRS UMR 6307, Inserm U 1200, Dijon, France

**Keywords:** General practitioners, Discrete choice experiment, Mixed logit, Latent class logit, Quality improvement programs, Policy simulation, I11, I18, C25

## Abstract

Despite increasing popularity, quality improvement programs (QIP) have had modest and variable impacts on enhancing the quality of physician practice. We investigate the heterogeneity of physicians’ preferences as a potential explanation of these mixed results in France, where the national voluntary QIP – the CAPI – has been cancelled due to its unpopularity. We rely on a discrete choice experiment to elicit heterogeneity in physicians’ preferences for the financial and non-financial components of QIP. Using mixed and latent class logit models, results show that the two models should be used in concert to shed light on different aspects of the heterogeneity in preferences. In particular, the mixed logit demonstrates that heterogeneity in preferences is concentrated on the pay-for-performance component of the QIP, while the latent class model shows that physicians can be grouped in four homogeneous groups with specific preference patterns. Using policy simulation, we compare the French CAPI with other possible QIPs, and show that the majority of the physician subgroups modelled dislike the CAPI, while favouring a QIP using only non-financial interventions. We underline the importance of modelling preference heterogeneity in designing and implementing QIPs.

## Highlights

- We combine latent class and mixed logit models to study heterogeneity in general practitioners’ preferences elicited from a discrete choice experiment
- We demonstrate that general practitioners exhibit substantive heterogeneity in preferences for quality improvement programs, notably for pay-for-performance
- We show that the majority of physicians dislike the implemented pay-for-performance program, and would favour non-financial interventions

## Background

Quality improvement programs (QIP) are an increasingly popular approach for enhancing the quality of physician practice in ambulatory care [1–3]. However, available evidence suggests that QIPs, whether they focus on or combine financial, non-financial or organizational components, have modest and variable impacts on quality of care [4–6]. Beyond methodological differences in the studies, this observed heterogeneity results from the target and design of the QIPs, as well as from variability in physicians’ responsiveness to the programs [7–10]. Within a single program, differences in physicians’ reactions may be explained by differences in contextual constraints, as well as knowledge or attitudes regarding the QIP [9, 10].

Physicians’ preferences for QIP are particularly important given that, in many cases, physicians’ participation is voluntary and, thus, necessary to ensure the success of the program. From 2009 to 2011, the French Statutory National Health Insurance implemented a voluntary QIP program (Contract for Improved Individual Practice – CAPI) aimed at general practitioners (GP), which combined pay-for-performance (P4P) and quarterly performance feedback. While the program could only increase their income, only one-third of all French GPs had registered a year and a half after the program’s implementation, and the program was subsequently cancelled due to its unpopularity^1^. While GPs’ ethical concerns with the program design was one key explanation of the low take-up of the CAPI [11], a QIP better designed to meet physicians’ work-related needs may have been more successful.

Health economists have thoroughly studied physicians’ preferences regarding their job characteristics [12, 13], sometimes accounting for preference heterogeneity [14–16]. Yet, no studies, to the best of our knowledge, have specifically examined physicians’ preferences for QIPs and their components. While recent studies have focused on designs of QIPs that would be effective irrespective of the targeted physicians [6, 17, 18], understanding these physicians’ preferences may allow for fine-tuning of the programs and improve acceptance. Moreover, understanding the heterogeneity of physicians’ preferences about QIPs may help policymakers tailor and diversify their programs to better match the needs of their targeted population.

The objectives of this study are precisely to elicit heterogeneity in physicians’ preferences for the components of QIPs; and by policy simulation, to compare the potential and differential impact on physician welfare of various QIPs, including the French CAPI. To do so, we conduct a discrete choice experiment (DCE) on a sample of French GPs.

## Methods

### Data and the discrete choice experiment

#### DCE design

Discrete choice experiments are widely used in the health economics literature to assess preferences [19]. Our study followed the recommended steps [20] as described below.

The first step of a DCE is to select the attributes of interest and their levels. We selected attributes based on a literature review on QIPs and on two criteria: supposed efficacy suggested by the literature and credibility of application in the French health care context (see Table 1). For concreteness, we focused on preventive care, a key quality indicator. Following the same two above criteria, a level for each attribute was defined to reflect the CAPI. The relevance of the list of attributes, of their number and of their levels was confirmed in a focus group of ten representative GPs [21]^2^. This led to a final list of eight attributes presented in Table 2.

**Table 1:** Interventions used in quality improvement programs for GPs

Component of the QIP	Justification
Financial component	
Amount of payment	The literature suggests a threshold of 5 % of the doctors’ income as a minimum for the incentive to be effective [52].
Method of remuneration	Financial incentives can improve the quality of care, but depend on the method and frequency of payment [6, 53]^a^. The three remuneration methods used in France are pay-for-performance (P4P), fee-for-service (FFS) and a kind of partial capitation known as a *forfait* ^*b*^.
Non-financial component	
Clinical guidelines	The efficacy of clinical guidelines is ascertained [54]. However, the kind of guideline used matters, and guidelines to which individual clinicians have contributed may be more effective in changing their behaviour [55].
Feedback on activity	Performance feedback, where physicians get quantitative feedback relate to their practice, increases quality of care [56].
Continuing education	Participation in continuing education increases adherence to clinical recommendations [57].
Organisational component	
Type of practice	There is an association between group practice and better quality of care [58, 59].
Non-physician provider	Quality of care is improved by cooperation of GPs with non-physician providers such as nurses [60].

^a^This point is subject to debate. Another study finds no effect of the frequency of P4P [61]. However, representative GPs in the focus group cited the importance of this attribute

^b^The French *forfaits* are a partial capitation payment that represents a small part of GPs income (6 % of income [62] for certain patients (chronically ill) or for the coordination and continuity of care). They complement the FFS but are absolutely not designed as a major payment. For example, the GP receives 40 euros a year for following each patient classified by the health insurance plan as chronically ill (*forfait pour affection de longue durée (ALD)*). In comparison, sector 1 GPs are paid 23 euros for each consultation at the physician’s office

**Table 2:** List of attributes and levels

Attributes	Levels
Level of remuneration^a^(annual increase)	100 Euros 6100 Euros 12,100 Euros
Method of remuneration	Lump sum (*forfait*) Lump sum and fee-for-service Lump sum and pay-for-performance
Frequency of remuneration	Monthly Annually
Prevention clinical guidelines	None Participatory guidelines (participation in their definition and application) Pre-established guidelines (evidence-based application)
Feedback on preventive practices	Yes No
Continuing education in prevention	Yes No
Type of practice	Group of GPs Solo practice
Assistance by non-physician providers during preventive work	Yes No

^a^We retain three levels: 0, 5 and 10 %. It was not possible to propose a truly null amount, so an amount very close to zero was proposed. French physicians are not accustomed to thinking about their income in percentage terms, thus the payment attribute was proposed in raw of the average income (in euros) rather in relative terms (in percentage)

The second step is to combine attributes into choice sets. Most of time, the combination relies on experimental plan theory since a full factorial design implies proposing too many choices to respondents [22] – 864 scenarios in our case. Using JMP software, we generated an orthogonal design [23] that resulted in 24 scenarios and achieved the properties of orthogonality and level balance. All other analyses are done with STATA. In order to facilitate respondents’ choices, we relied on a common comparator selected from these 24 scenarios, ensuring that this reference scenario is not strictly dominant a priori [24]. Choice sets were constructed by pairs which resulted in 23 choices between pairs of combinations of quality interventions. The 23 choice sets were randomly divided into four blocks so that each respondent made 5 or 6 choices [25]^3^. To limit non-response and the subsequent loss of statistical efficiency, we did not include an opt-out possibility. An example of choice set is provided in Appendix 1.

Finally, the DCE was pilot tested with a focus group of self-employed GPs to validate the attributes phrasing and then pre-tested (*n* = 100 GPs) to verify that the reference scenario was not strictly dominant.

#### Data

The DCE questionnaire is composed of three parts. In the first part, questions regarding the GP’s opinion about health care reforms in general practice and the public health role of GPs are used as a warm-up. The second part is the choice experiment. The third part collects sociodemographic and professional information about each GP. The questionnaire is self-administered during the summer of 2009 in a postal survey with one repeated attempt for non-response.

The population under study consists of all the GPs in active practice in one French geographic region^4^ (*N* = 1368). After the pre-test, the questionnaires were sent to the 1268 remaining physicians. 303 questionnaires were returned completed, resulting in a response rate of 22 %. This response rate is consistent with other DCE studies [26–28] and with self-administered postal surveys to French general practitioners [29].

GPs working in a rural setting are slightly overrepresented in our sample (see Table 3). The responding GPs are also more active, with the weekly number of acts being significantly higher than the national mean^5^. With these exceptions, our sample compares well with the reference population. Of course, our methodology does not allow for national representativeness.

**Table 3:** Descriptive statistics

Variables	Sample (*N* = 303)	Mean value in Bourgogne	Difference sample and regional (*p*-value)	Mean value in France	Difference sample and national (*p*-value)
Age (mean)	51.5	51.2^(a)^	0.451 (n.s)	51.3^(a)^	0.588 (n.s.)
Gender (% of women)	27 %	30 %^(c)^	0.479 (n.s.)	31.2 %^(b)^	0.277 (n.s.)
Sector of activity (% in sector 1)	93.1 %	87.3 %^(a)^	0.485 (n.s.)	89.3 %^(a)^	0.623 (n.s.)
Rural practice (%)	44.5 %	33 %^(d)^	0.000	15.7 %^(b)^	0.000
Group practice (%)	47.5 %	39.6 %^(d)^	0.118 (n.s.)	44.5 %^(b)^	0.567 (n.s.)
Health network membership (%)	41.9 %	39 %^(e)^	0.496 (n.s.)	Between 27 and 44 % (5 French region)^(e)^	Not determined
Weekly acts (mean)	119	102.8^(a)^	0.000	102.4^(a)^	0.000

In the absence of exhaustive and homogeneous data source on private practice self-employed GPs, the regional and national values are derived from different sources

^a^All private practice GPs – 2008 data – SNIIR – source: Eco-Santé France, Régions & Départements 2015 – IRDES [63] (for the weekly activity, the number of annual acts has been divided by 46 weeks)

^b^All private practice GPs –2009 data – ADELI – [64]

^c^All private practice GPs –2009 data – SNIIR – [65]

^d^Survey panel of five regions (*panel de médecins généralistes libéraux DREES, URML, FNORS*) – 2007 data – [66]

^e^Survey panel of five regions (*panel de médecins généralistes libéraux DREES, URML, FNORS*) – 2007 data – [67]

With the exception of the level of remuneration, all attributes of the DCE are coded using “effects coding” [30]. We constructed the questionnaire in order to test the symmetry [31], the completeness and the continuity axioms [32]^6^ and found that the axioms are largely respected: totally for the first, and respectively by 82 % and 65 % of the respondents for the two other axioms. Following current practice, we kept all the responses for the analysis [32–34].

### Econometric framework

#### Modelling heterogeneity

The analysis of DCE data relies on classical choice models and random utility theory (RUT) [35]. When applying the DCE approach, the utility of an individual *n* choosing alternative *i* at the *t* choice situation can be written as

$$ {U}_{nit}={V}_{nit}+{\varepsilon}_{nit} $$

Where ${V}_{nit}={\displaystyle {\sum}_{k=1}^K}{\beta}_kx{\hbox{'}}_{nitk}$ is the deterministic part of the utility (with *k* attributes), observable to the researcher and sometimes referred to as the indirect utility, and *ε*_*nit*_ is the unobservable, stochastic part and is treated as random^7^. The individual will choose the alternative yielding the highest utility.

The conditional logit is the most commonly used method to analyse DCE data, but relies on restrictive assumptions on the stochastic terms [23], fails to incorporate the panel structure of most DCE data and does not account for preference heterogeneity. The two principal models that circumvent these limitations are the mixed logit (MXL) [36, 37] and the latent class model (LCM) [38].

The choice between these two models critically depends on expectations about the variation of preferences [39]: if researchers expect preferences to vary greatly between individuals, the MXL is preferred; the LCM is preferred if individuals are thought to be grouped in homogeneous latent groups. However, the information the models provide is complementary: MXL provides information about how heterogeneity is distributed relative to each attribute while LCM informs on the heterogeneity among latent subgroups of physicians. Thus, we elect to run both MXL and LCM.

The unconditional probability of a mixed model that allows for individual-specific variation in tastes and accounts for the panel dimension of choices is as follows [40]:

$$ {P}_{nI}\left(\theta \right)={\displaystyle \int }{S}_{nI}f\left(\beta \Big|\theta \right)d\beta $$

Where ${S}_{nI}\left(\beta \right)={\displaystyle {\prod}_{t=1}^T}\left[\frac{ \exp \left(\beta \hbox{'}{x}_{nit}\right)}{{\displaystyle {\sum}_{j=1}^J} \exp \left(\beta \hbox{'}{x}_{njt}\right)}\right]$ is the conditional probability that the individual *n* realises a choice sequence *I* = {*i*_1_, …, *i*_*t*_}, *f*(*β*|*θ*) is a density function of the individual-specific *β* with distribution parameters *θ* (see [40] for more on the family of mixed models).

Preference heterogeneity is reflected in the density function, *f*(*β*|*θ*), and the distribution of *β* can be either continuous or discrete, implying MXL or LCM, respectively.

The other major difference between the models is the estimation method. Each model relies on log-likelihood maximization, with the log-likelihood given by $LL\left(\theta \right)={\displaystyle {\sum}_{n=1}^N} ln{P}_n\left(\theta \right)$. Unlike the LCM, this expression cannot be solved analytically in MXL and simulation methods are used for approximation [38, 40].

#### Simulating policy

The goal of the policy simulation is to evaluate the effects of changes in the three main components of a QIP (financial, non-financial and organizational), and we use the compensating variation (CV) method to measure the relative impact on GPs’ welfare of such change [41, 42].

The CV is calculated using the utility estimates computed after the regressions in the following expression [41]

$$ CV=-\frac{1}{\beta_w}\left[ ln{\displaystyle {\sum}_{j=1}^J} \exp \left({V}_j^0\right)- ln{\displaystyle {\sum}_{j=1}^J} \exp \left({V}_j^1\right)\right] $$

Where *β*_*w*_ is the marginal utility of income, ${V}_j^0$ is the indirect utility for each option *j* before the policy change and ${V}_j^1$ the same after the policy change. In our case, we consider only two policy options at a time, the CAPI versus something else. The formula is then simplified to [20]

$$ CV=-\frac{1}{\beta_w}\left[{V}_j^0-{V}_j^1\right] $$

The question of heterogeneity is evaluated by estimating CV for each latent group of physicians with LCM. For MXL, we compute and compare CV for the specific attributes where GPs exhibit significantly heterogeneous preferences (*e.g.* those GPs obtaining positive versus negative marginal utility from the attribute).

#### Model specification

We include an intercept in all models. This alternative-specific constant (ASC) is necessary since choices are made relative to a fixed comparator (the constant scenario) [30, 42]. In our case, this ASC has no natural interpretation and is expected to be statistically insignificant [12].

When specifying a mixed logit it is critical to choose which parameters are allowed to vary and which distribution these latter will follow. The normal and log-normal distributions are the most commonly used for the random coefficients [39, 40, 43]. As the log-normal distribution is criticised for its long right tail [37, 44], we choose the normal distribution^8^.

The possibility to specify the coefficients as random is one of the great strengths of the MXL. The ASC is fixed since it has no reason to vary between the respondents. Fixing the monetary attribute (the remuneration) has several advantages [45]. In our case, the main one is the capacity to calculate CV. The possibility of significant preference heterogeneity in terms of remuneration cannot be ruled out and should be considered in order to fully understand physicians’ preferences. GPs valuing less payment can indeed be explained in an intrinsic motivation framework, among others. We therefore run two MXL: one with all coefficients normally distributed except the constant and the amount of remuneration coefficient (MN1) and the other with only the constant term fixed (MN2).

Without an intuitive way to choose the number of latent classes in LCM, the decision is often made on the basis of goodness-of-fit measures [27, 39]. We use the Akaike (AIC), Bayesian (BIC) and consistent Akaike (CAIC) information criteria.

The results for the selection of the number of classes are presented in Table 4. The BIC and CAIC show that the best fit is obtained with four classes, a number we retain for the following analyses^9^.

**Table 4:** Selection of the number of classes for the LCM

	AIC	BIC	CAIC	Log likelihood
2 classes	1885.706	1971.1219	1994.1219	-919.8531
3 classes	1859.6501	1989.6307	2024.6307	-894.8251
4 classes	1783.5122	1958.0576	2005.0576	-844.7562
5 classes	1780.0136	1999.1239	2058.1239	-831.0069
6 classes	1787.6742	2051.3492	2122.3492	-822.83712

## Results

### Heterogeneity in GPs’ preferences

The estimation results for the mixed logit are presented in Table 5. The sign, significance and magnitude of the mean coefficients are very stable between the two models (MN1 and MN2), underlining the robustness of the results. The ASC is not significant, indicating that respondents have made their choice only on the basis of the attributes in the list (so the model is correctly specified). The estimates reveal the existence of preference heterogeneity among GPs that is quite concentrated around some attributes.

**Table 5:** Estimation of the mixed logit models

		MN1		MN2	
		Coefficient	t-Stat	Coefficient	t-Stat
Level of remuneration	Mean	0.0002***	(9.03)	0.0002***	(6.59)
	SD	-	-	0.0003***	(7.21)
*Forfait*	Mean	-0.4706*	(-2.41)	-0.6635*	(-2.57)
	SD	0.1203	(0.41)	0.1227	(0.34)
Pay-for-performance	Mean	-0.5085*	(-2.36)	-0.6608*	(-2.38)
	SD	0.9771***	(5.06)	1.2575***	(6.13)
Frequency	Mean	0.2652	(1.66)	0.3264	(1.69)
	SD	0.0782	(0.40)	0.2098	(1.00)
Definition of guidelines	Mean	0.4966*	(2.35)	0.6776*	(2.55)
	SD	0.2992	(0.97)	0.0796	(0.36)
Application of guidelines	Mean	0.2563	(1.24)	0.3396	(1.27)
	SD	0.1060	(0.33)	0.5811*	(2.06)
Continuing education	Mean	0.6580***	(3.89)	0.8654***	(4.39)
	SD	0.3710	(1.11)	0.0312	(0.11)
Information feedback	Mean	0.4070*	(2.07)	0.4801*	(2.06)
	SD	0.4751	(1.78)	0.1112	(0.30)
Solo practice	Mean	0.3476*	(2.19)	0.4902**	(2.61)
	SD	0.2119	(0.82)	0.4721*	(2.23)
Assistance by NPP	Mean	0.1057	(0.61)	0.1641	(0.78)
	SD	0.9063***	(5.72)	1.2831***	(7.45)
ASC	Mean	1.3462	(1.40)	1.7370	(1.51)
Number of observations		3390		3390	
Number of respondents		303		303	
Log Likelihood		-908.4154		-879.5045	
AIC		1856.8309		1801.0089	
BIC		1979.4026		1929.7092	

*Significant at 5 %; **significant at 1 %; ***significant at 0.1 %

The standard deviations are significant for the pay-for-performance and the assistance by NPP in model MN1. In MN2, this is also the case for the application of guidelines, the type of practice, and the level of remuneration. The heterogeneity in preferences for pay-for-performance is particularly relevant. This remuneration scheme is a source of marginal disutility at the mean but is positively valued by 22 % and 24 % of physicians (in MN1 and MN2, respectively). These figures are consistent with the proportion of French GPs having chosen to adhere to the CAPI (around 30 %, [11]). It is also worth noting that the indifference to the assistance by NPP at the mean masked a strong heterogeneity. Indeed, 60 to 62 % would like to benefit from this kind of assistance. Finally, even the amount of remuneration is marked by heterogeneity, with 14 % of physicians not valuing an increase in income for the targeted activities (MN2).

The latent class model estimates are presented in Table 6. Over all the classes, the ASCs are insignificant. For the first class, the only significant attributes are continuing education and assistance by NPP. Continuing education has a positive effect on indirect utility while assistance by NPP has a negative one. In the second class, the significance of the attributes is slightly different. While continuing education remains significant, this time it has a negative effect. GPs in this class prefer higher payment and to be paid more often, as the sign and significance of the frequency attribute attests. They dislike the *forfait* but they are indifferent to pay-for-performance. They also prefer solo practice. All attributes are significant for classes 3 and 4, however distinct behaviour is observed. The doctors in these two latent classes place negative value on alternative payment relative to FFS while preferring more frequent payment. They also prefer to work in groups. They differ in respect to all the other attributes. In contrast to the third class, an increase in remuneration has a negative effect on indirect utility in the fourth class. Class 3 physicians disvalue all types of clinical guidelines but positively value continuing education and information feedback, contrary to class 4. Physicians in the fourth class value assistance by NPP while those in the third class do not. With the preference for group practice in both classes, this result suggests a preference for physician groups only in class 3 while multidisciplinary teams are preferred in class 4.

**Table 6:** Estimation of the latent class logit model – 4 classes

	Class 1		Class 2		Class 3		Class 4	
	Coefficient	t-Stat	Coefficient	t-Stat	Coefficient	t-Stat	Coefficient	t-Stat
Level of remuneration	-0.0001	(-1.56)	0.0002***	(7.35)	0.0023***	(19.04)	-0.0030***	(-18.85)
*Forfait*	-0.2202	(-0.35)	-0.8085*	(-2.07)	-9.6873***	(-14.26)	-26.8455***	(-18.89)
Pay-for-performance	-1.5179	(-1.94)	0.6209	(1.71)	-24.0380***	(-29.94)	-20.2301***	(-15.56)
Frequency	-0.5197	(-0.84)	0.9612**	(2.70)	1.2295*	(2.31)	34.5608***	(34.54)
Definition of guidelines	1.8732	(1.76)	-0.1382	(-0.41)	-3.8807***	(-6.19)	46.0073***	(27.24)
Application of guidelines	2.0941	(1.79)	0.5921	(1.70)	-14.4822***	(-22.98)	17.9852***	(19.47)
Continuing education	3.6665***	(4.51)	-1.0573**	(-3.13)	11.0212***	(14.31)	-6.5797***	(-5.23)
Information feedback	-0.6791	(-1.29)	0.1495	(0.37)	7.4359***	(8.38)	-4.5607***	(-4.39)
Solo practice	-1.2745	(-1.63)	1.0318***	(3.44)	-3.9727***	(-7.85)	-8.1784***	(-9.51)
Assistance by NPP	-1.3672*	(-2.32)	0.4714	(1.88)	-11.7878***	(-17.33)	40.3411***	(36.88)
ASC	2.8629	(0.54)	2.7353	(1.00)	-67.6568	(.)	72.4956	(.)
Average class share	0.136		0.317		0.231		0.316	
Number of observations	3390							
Number of respondents	303							
Log Likelihood	-844.7561							
AIC	1779.5122							
BIC	2055.2985							

*Significant at 5 %; **significant at 1 %; ***significant at 0.1 %

At this point it is worth comparing the results of the two kinds of models. One of the major conclusions, holding in both MXL and LCM, is the negative impact on indirect utility of an increase in remuneration observed for some GPs. It shows that this result is not only a matter of statistical artefact resulting from the use of a normal distribution in the MXL [39]. The MXL underlined heterogeneity of preferences for P4P. This heterogeneity is also found in the LCM, with the third and fourth classes disliking this payment while the coefficient is positive in the second class (but significant only at 10 %). The strong difference in preferences for assistance by NPP found in MXL is also seen in LCM. The negative coefficients in classes 1 and 3 are contrasted by a strong positive preference in class 4. All in all, this suggests a stability of the main conclusions made from the different models, with preference heterogeneity remaining among classes.

Regarding the goodness of fit of the models, results in Table 7 indicate very little advantage to LCM while MXL (MN2) has better BIC. The minimal difference between the best fitting models suggests that each provides relevant information on the heterogeneity of GPs’ preferences.

**Table 7:** Goodness-of-fit measures of the different specifications

	AIC	BIC	Log likelihood
MN1	1854.961	1977.532	-907.4804
MN2	1794.699	1923.4	-876.3496
LCM(4)	1783.5122	1958.0576	-844.75616

### Simulating alternative quality improvement programs

The policy simulation study relies on the calculation of compensating variation. The goal is here to evaluate the relative impact on physicians’ welfare of alternative QIPs to the CAPI. These alternatives were chosen to be consistent with, and believable in, the context of French general practice.

The DCE attributes are used to depict five QIPs – the CAPI and four alternative policies (refer to Appendix 2 for more details). The first is close to the emerging organizational model in French primary care (*maisons pluridisciplinaires et pôles de santé*) implemented to foster quality of care, and also known in the literature as “integrated” primary care model [46]. The second introduces a mixed remuneration scheme that can better balance quantity and quality in physicians’ activity [47]. In order to measure only the effect of the payment scheme, we assume an increase in income similar to the CAPI. The third QIP is composed of only non-financial mechanisms that do not require a sharp transformation in physicians’ organization (i.e. no multidisciplinary team). The fourth is designed as a maximal satisfaction policy and is used as a benchmark^10^. Even if the maximum satisfaction of GPs is not necessarily an objective *per se*, comparing it to the CAPI gives a sense of the distance separating this QIP from the most desirable one. The details of each policy are presented in Table 8.

**Table 8:** CAPI and alternative QIPs

	CAPI	Integrated primary care model (P1)	Mixed remuneration (P2)	Non-financial interventions (P3)	Maximum satisfaction (P4)
Level of remuneration	4200	4200	4200	0	4200
Method of remuneration	*Forfait* and P4P	*Forfait*	*Forfait* and FFS	No	*Forfait* and FFS
Frequency of remuneration	Annual	Annual	Annual	No	NA
Prevention clinical guidelines	No	Pre-established	No	Participatory	Participatory
Continuing education in prevention	No	Yes	No	Yes	Yes
Feedback on preventive practices	Yes	Yes	No	Yes	Yes
Group practice	No	Yes	No	No	No
Assistance by non-physician providers	No	Yes	No	No	NA

In the last column, the frequency of remuneration and assistance by NPP are not considered because GPs are indifferent to it at the mean. The maximum satisfaction is defined for all GPs. The French *forfait* are paid annually per patient (P1). FFS means a payment at each visit and cannot be “monthly” or “annual”, but mixed remuneration here includes a *forfait*, so we select the annual frequency for P2

The indirect utilities and the corresponding CV are first computed for all GPs on the basis of MN1 estimates. With mixed logit models, we concentrate on the attributes which are consistently heterogeneous in the two models (MN1 and MN2): P4P and assistance by NPP. For each, we identify “inclined” who obtain positive marginal utility from these attributes and “adverse” who obtain negative marginal utility. The LCM provides natural subgroups for the estimation of CV, which are computed in the four latent classes. It should be noted that only the significant coefficients enter in the computation of CV for each subgroup of interest. As GPs are indifferent to insignificant attributes, using their estimate values would distort the welfare estimates. Results are presented in Table 9.

**Table 9:** Policy simulation: compensating variation (Euro per year)

		CAPI	Integrated primary care model	Mixed remuneration	Non-financial interventions	Maximum satisfaction
All GPs	Indirect utility	-0.5143	0.8974	0.1594	1.9093	3.5391
	CV	x	9113	4349	15646	26167
P4P “inclined”	Indirect utility	0.1824	1.0987	-0.7463	2.1993	3.1624
	CV	x	5915	-5995	13020	19238
P4P “adverse”	Indirect utility	-0,7121	0,8542	0,4087	1,8295	3,6504
	CV	x	10112	7236	16408	28163
Assistance by NPP “inclined”	Indirect utility	-0.7463	1.4502	-0.6057	1.6088	3.0452
	CV	x	14180	908	15204	24477
Assistance by NPP “adverse”	Indirect utility	-0.3814	0.2714	1.1420	2.1565	4.1030
	CV	x	4214	9835	16384	28951
Class 1	Indirect utility	-1.9848	2.6139	-1.9848	5.0337	5.3483
	CV	x	61397	0	93705	97905
Class 2	Indirect utility	2.0676	-2.9193	2.0676	-0.0255	0.9140
	CV	x	-22293	0	-9357	-5157
Class 3	Indirect utility	7,0764	-5,0059	49,9679	22,3915	65,8679
	CV	x	-5204	18474	6596	25323
Class 4	Indirect utility	-152.8812	6.3605	-76.4541	-13.6526	45.8256
	CV	x	53925	25881	47148	67290

The first striking result is that CAPI is a source of indirect disutility in the majority of the subgroups considered (5 out of 8).

The compensating variation indicates the annual benefits for GPs of choosing an alternative QIP rather than the CAPI. P4P “inclined” have a positive indirect utility from the CAPI of course. However, with the exception of the mixed remuneration program, all other alternative policies still give a greater benefit than the CAPI^11^. P4P “adverse” would prefer each of the alternative policies to the CAPI, if they were proposed. The non-financial policy has the greatest CV, but the gap with integrated primary care is reduced. Whether they are “inclined” or “adverse” to assistance by NPP, GPs disvalue the CAPI and prefer all alternatives. We expected the NPP “inclined” to have a greater benefit from P1 because of the multidisciplinary team but P3 is a little more valued,. The NPP “adverse” have their lowest (though still positive) CV for P1 and their preferred alternative is the non-financial program P3.

The patterns are very different between latent classes. Classes 1 and 4 obtain negative and extremely negative indirect utility from the CAPI, respectively, while the sign is positive in classes 2 and 3. Compared to the other subgroups, CV is very high in class 1^12^. The benefit of having the non-financial policy rather than the CAPI is equivalent to 93,705€, almost the same amount as for the maximum satisfaction program. There is no benefit from shifting from the CAPI to the mixed remuneration scheme. This last result holds for class 2. This class is very specific since it is the only subgroup where other policies result in losses. It is even the case for P4, designed to be the most desirable for GPs in the whole, underlining again the particularity of this latent group. For class 3, mixed remuneration has the highest CV, with a relative benefit of 18,474€. With the exception of P1, alternative policies still dominate the CAPI. For class 4, integrated primary care offers the highest relative benefit (53,925€) while the CV for the non-financial policy remains important (47,148€).

## Discussion and conclusion

Using a discrete choice experiment, we elicited French GPs’ preferences for the different components of QIPs. We showed the strength of heterogeneity in their preferences and demonstrated how this heterogeneity leads physicians to evaluate very differently the same interventions aimed at improving the quality of care. The heterogeneity in preferences is concentrated on some components, especially P4P and assistance by a NPP. There is also variation in preferences by latent groups of GPs, with some physicians valuing some components of QIP only (continuing education and assistance by NPP in group 1), while other physicians value the same components differently (group 3 versus 4). Given this heterogeneity, the crucial policy lesson is that QIPs could be adapted to meet physicians’ preferences by offering a menu of programs and allowing GPs to self-select. If policymakers were to choose only one QIP, CV indicates that they should implement a program using only non-financial interventions. Yet, policymakers continue to rely heavily on the financial dimension to change physician behaviour with QIP, as it is the case in France with the ROSP – the QIP that has replaced the CAPI. Strong beliefs in the power of the financial lever or perceptions of potential implementation difficulties for non-financial interventions could explain this policy choice. Another interpretation is that financial QIP could be seen as a mechanism to both address unavoidable compensation claims from medical union and concerns for the quality of care.

Some limitations should be noted. First, the limited response rate, though consistent with the DCE literature, may have led to sample selection bias. While we do not have information on the non-responders, the opinions expressed in the first part of the questionnaire are reassuring in the sense that they are quite close to those expressed in other French studies [48–50]. Second, the use of a forced choice design might have biased the estimates if physicians wished to choose neither of the two proposed QIP. However, physicians who were not willing to choose one of the two options in a given choice set actually did not respond at the specific choice occasion, the forced choice is still used in health professional DCE studies [15], and this “forced choice” strategy is consistent with the new orientation of the French national QIP program (the ROSP is mandatory). Finally, we choose to use a common comparator when we constructed the choice set, which does not necessarily maximize the statistical efficiency of the experimental design [22]. Yet, fixed comparator increases the “respondent efficiency”, which can be defined as the capacity of a respondent to express his “real” preferences in the context of the DCE [51]. Given that private practice physicians are heavily time-constrained, particularly in the French fee-for-service context, we believe this trade-off between statistical and respondent efficiency has allowed us to obtain a satisfactory response rate and better quality and completeness of responses relative to other designs.

Despite these limitations, this study adds to the broader literature on the heterogeneity of health professionals’ preferences [13–15, 28] and for the first time, combines LCM and MXL approaches. Each model contributes a better understanding of physicians’ preferences and using such an approach can help policymakers to better design their QIP.

## Appendix 1

**Table 10:** Example of choice set

	Option A	Option B
Income increase per year	6100€	12100€
Method of remuneration	*Forfait* + Fee-for-service	*Forfait* + Pay-for-performance
Frequency of remuneration	Annually	Annually
Work in group of general practitioners	Yes	No
Prevention clinical guidelines	None	You participate in their definition and application
Continuing education in prevention	No	Yes
Feedback on preventive practices	No	No
Assistance by non-physician providers during preventive work	No	Yes
	I prefer A	I prefer B
*Tick one box*	□	□

Note: Translated from French

## Appendix 2

**Table 11:** Construction of the CAPI scenario

Attributes	Level	Justification
Level of remuneration	4200	The maximum bonus a GP can earn is 7000€ a year, from which only 60 % is imputable to preventive services. We select this maximum in order to evaluate the highest benefit that can be expected from the CAPI.
Method of remuneration	*Forfait* and Pay-for-performance	The CAPI introduced P4P in France. A *forfait* per patient is adjusted depending on the attainment of the clinical practice targets.
Frequency of remuneration	Annual	The payment is made at each anniversary of the signed contract.
Prevention clinical guidelines	No	Even though various guidelines exist, they are not linked with the CAPI.
Feedback on preventive practices	Yes	Information is fed back to the doctor each trimester as part of the CAPI.
Continuing education in prevention	No	Continuing education is only on a voluntary basis and is not linked to the CAPI.
Group practice	No	No incentive for GPs working in teams is included in the CAPI.
Assistance by NPP	No	Assistance by NPP is not provided or supported under the CAPI.

## Footnotes

1 The CAPI was replaced in 2012 by a P4P program (the ROSP) where physicians are enrolled automatically, but can request to opt out.

2 The number of considered attributes should not be so high as to allow respondent to make trade-offs. If there are no clear recommendations on the maximum number, the DCE health economics literature generally uses at most eight attributes [21].

3 Caussade et al. [25] showed that setting between 6 and 13 choice situations minimizes the error variance of the estimates.

4 We restricted ourselves to the region of Bourgogne because of prior relationships with the regional health professional’s union who facilitated the constitution of the focus-group and offered logistic support for the survey. The restriction to one region is also due to monetary and time constraints. The fund obtained from the *Conseil Regional de Bourgogne* did not allow for a survey of more than one region.

5 This point is, however, not particularly concerning as the regional and national values are derived from an administrative database (*système national d’information inter-régimes* – SNIIR) known to underestimate physicians’ activity. The SNIIR includes the very low activity physicians, pulling down the average number of acts.

6 Beyond the choice exercise, supplementary choices and follow-up questions were introduced in the DCE in order to test the internal validity of the data collected. More information on the test procedures used is available upon request.

7 This random part is precisely why RUT can deal with axiomatic violations. The “errors” may come from this stochastic part, which is unexplained by the researcher.

8 Because of the qualitative nature of the majority of our attributes, there is no reason to think that one level should be preferred to another a priori. It is therefore difficult to select the sign of the distribution. MXL with log-normal distribution are run for sensitivity analyses and do not exhibit large differences in the fit. Results are available from the authors.

9 The simplest way to account for heterogeneity of preferences is to incorporate the personal characteristics of the respondents in the models. It can be done with interaction terms in the MXL and to explain class membership probability in the LCM. We argue these individual characteristics have to considerably improve the fit of the models in order to be worth keeping for final analysis, which is not the case with our data (results available upon request). Most of the personal characteristics are found to be insignificant in the MXL, the integration of these characteristics does not drastically improve the fit to the data in MXL, and even worsens the fit of the LCM. As a result and following Hole [39], we focus only on the more parsimonious models in our analyses.

10 The maximal satisfaction policy is designed for all GPs. Our goal is to compare different nationally uniform policies such as the CAPI.

11 In this paragraph, when talking about greater CV, we do not take the maximum satisfaction into account and only concentrate on the reliably implantable policies.

12 This is partly because only two attributes are found significant for this latent class and enter the calculation of the CV. Their presence or absence therefore has a disproportionate impact on the CV for each policy.
